# Comparison of Methods for Quantification of Global DNA Methylation in Human Cells and Tissues

**DOI:** 10.1371/journal.pone.0079044

**Published:** 2013-11-18

**Authors:** Sofia Lisanti, Wan A. W. Omar, Bartłomiej Tomaszewski, Sofie De Prins, Griet Jacobs, Gudrun Koppen, John C. Mathers, Sabine A. S. Langie

**Affiliations:** 1 Human Nutrition Research Centre, Institute for Ageing and Health, Newcastle University, Campus for Ageing and Vitality, Newcastle upon Tyne, United Kingdom; 2 Centre for Integrated Systems Biology of Ageing and Nutrition, Institute for Ageing and Health, Newcastle University, Campus for Ageing and Vitality, Newcastle upon Tyne, United Kingdom; 3 Environmental Risk and Health unit, Flemish Institute for Technological Research (VITO), Mol, Belgium; 4 Advance Medical and Dental Institute, Universiti Sains Malaysia, Penang, Malaysia; 5 Centre for Brain Ageing and Vitality, Institute for Ageing & Health, Newcastle University, Campus for Ageing and Vitality, Newcastle upon Tyne, United Kingdom; 6 Faculty of Pharmaceutical, Biomedical and Veterinary Sciences, University of Antwerp, Antwerp, Belgium; University of Bristol, United Kingdom

## Abstract

DNA methylation is a key epigenetic modification which, in mammals, occurs mainly at CpG dinucleotides. Most of the CpG methylation in the genome is found in repetitive regions, rich in dormant transposons and endogenous retroviruses. Global DNA hypomethylation, which is a common feature of several conditions such as ageing and cancer, can cause the undesirable activation of dormant repeat elements and lead to altered expression of associated genes. DNA hypomethylation can cause genomic instability and may contribute to mutations and chromosomal recombinations. Various approaches for quantification of global DNA methylation are widely used. Several of these approaches measure a surrogate for total genomic methyl cytosine and there is uncertainty about the comparability of these methods. Here we have applied 3 different approaches (luminometric methylation assay, pyrosequencing of the methylation status of the Alu repeat element and of the LINE1 repeat element) for estimating global DNA methylation in the same human cell and tissue samples and have compared these estimates with the “gold standard” of methyl cytosine quantification by HPLC. Next to HPLC, the LINE1 approach shows the smallest variation between samples, followed by Alu. Pearson correlations and Bland-Altman analyses confirmed that global DNA methylation estimates obtained via the LINE1 approach corresponded best with HPLC-based measurements. Although, we did not find compelling evidence that the gold standard measurement by HPLC could be substituted with confidence by any of the surrogate assays for detecting global DNA methylation investigated here, the LINE1 assay seems likely to be an acceptable surrogate in many cases.

## Introduction

DNA methylation is a chemical modification of the genome which involves the covalent addition of a methyl group to DNA, mainly occurring at cytosine residues within CpG dinucleotides. Although in mammalian genomes the CpG dinucleotide is under-represented, unusually dense clusters of CpG dinucleotides called “CpG islands” are present [1] and overlap promoter regions of 50%–60% of human genes [2], thus suggesting that DNA methylation plays a role in the regulation of gene expression. Nonetheless most of the DNA methylation in mammalian genomes is found in repetitive elements, such as transposons and endogenous retroviruses [3]. Transposable elements, which include long terminal repeats (LTR)-retrotransposons, long and short interspersed nuclear elements (LINE and SINE, respectively) and DNA transposons constitute about 45% of the human genome. These sequences could interfere with the regulation of gene expression and genome structure by means of insertions, deletions, inversions and translocations of genomic sequences. However, this potential damage is decreased by high levels of CpG methylation which effectively silences these repetitive regions [3], [4].

Altered global DNA methylation content is a feature of several diseases; its occurrence in human tumors was first highlighted by Gama-Sosa et al. [5]. Hypermethylation of the promoter regions of tumor suppressor genes and DNA repair genes, such as *MLH1,* causes gene silencing and contributes causally to tumorigenesis [6]. Similar gene silencing effects are seen in Fragile X Syndrome [7], [8]. Altered patterns of DNA methylation are observed during ageing, some of which correlate with age-related frailty and other age-dependent phenotypes [9]–[11]. Recent studies have identified cancer-specific differentially DNA-methylated regions in colon cancer [12] and other solid tumors including lung, breast, thyroid and Wilms' tumors [13]. These cancer-specific changes include hypomethylation of approximately 50% of the genome which was associated with high levels of variability in gene expression [13].

Several approaches have been developed which are regarded as gold standard methods for measurement of global methylation (e.g. high performance liquid chromatography (HPLC) [14] or variants of this approach such as HPLC tandem mass spectrometry (LC-MS/MS) [15], as well as two dimensional thin layer chromatography [16] and high performance capillary electrophoresis [17]). After DNA digestion, in chromatographic approaches e.g. HPLC, the single nucleotides are separated according to size and both cytosine and methylated cytosine are quantified [14]. Whilst this method is highly quantitative and reproducible, it requires relatively large amounts of DNA and the protocol for assay optimization is demanding. Thus other methods to estimate global methylation content that require less DNA and use more readily available equipment have been developed. These include PCR based methods which estimate the methylation status of major genomic repeat elements e.g. Alu and LINE1 [18] and methylation sensitive restriction assays such as the luminometric methylation assay (LUMA) [19], [20].

In the Alu and LINE1 assays, the methylation status of specific cytosine residues in bisulfite converted DNA is quantified, often by pyrosequencing. Since bisulfite treatment converts non-methylated cytosines to uracil residues that are converted to thymidine in a subsequent PCR reaction, the ratio of cytosine/converted thymidine residues is indicative of the methylation status of the sequence of interest. In the LUMA assay, DNA samples are digested in parallel with the isoschizomers MspI (unaffected by methylation) and HpaII (methylation sensitive), which recognize the same sequence (CCGG), and cut differentially according to the methylation state of the internal cytosine residue. The digestion ratio HpaII/MspI can be determined by pyrosequencing and the resulting ratio is inversely proportional to the methylation content of the sample.

Compared with HPLC, these methods may be less expensive and require less starting material, but they provide information on methylation levels limited to the specific analyzed sequences. Although these simplified approaches for global DNA methylation estimation are widely used as surrogates for total genomic DNA methylation, there is uncertainty about their comparability and the extent to which they reflect measurements of total methyl cytosine content of DNA.

In this paper, we applied three surrogate approaches (Alu, LINE1 and LUMA) for global DNA methylation estimation to the assay of a range of human cells and tissues, namely: HeLa cells (human cervical cancer cells) and M059J cells (human glioblastoma cancer cells) both untreated and treated with the demethylating agent 5-azacytidine, and human colon biopsies (matched normal mucosa and tumor tissue from the same patients). Our criteria for utility of individual approaches for estimating global DNA methylation included: i) ability to detect biologically important differences in tissue methylation (tumor vs. normal), ii) ability to detect effects of a demethylating agent (5-azacytidine), iii) concordance of results using each approach with data obtained by the gold standard assay (HPLC) and iv) variability of results, ease of use and relative costs.

## Materials and Methods

### Human colonic biopsies

Colon biopsies were collected from patients (n = 10) who underwent surgery for colorectal cancer at Wansbeck General Hospital (Ashington, Northumberland, UK). All of these patients gave written informed consent. Ethical approval was received from the Northumberland Local Research Ethics Committee (project reference NLREC2/2001). Samples of both normal mucosa (>10 cm from tumor margin) and matched tumor tissue from the same patient were collected in the operating theatre, immediately after tissue resection. All samples were snap-frozen immediately in liquid nitrogen and stored at −80°C. Details of this study have been published [21].

### Cell culture and 5-azacytidine (5-AzaC) treatment

HeLa and M059J cells were cultured in DMEM (Sigma) supplemented with 10% heat inactivated FCS and 1% penicillin/streptomycin. Cells were maintained at 37°C in a 5% CO_2_ atmosphere. To generate cells with different levels of DNA methylation, cells at approximately 50% confluency were treated with 5 μM of the demethylating agent 5-azacytidine (5-AzaC, Sigma). Treatment continued for 3 days, while replacing DMEM plus 5-AzaC daily. After treatment, cells were harvested, divided in 3 aliquots and frozen as cell pellets at −80°C.

### Various assays to measure global DNA methylation

Table 1 provides an overview of each of the individual approaches used in this study for estimating global DNA methylation, along with the biological relevance of the variable measured. Further details of these assays are given below.

**Table 1:** Overview of various assays to assess global DNA methylation, depicting their biological relevance.

Assay	Biological relevance	Amount of DNA required (used in our assay)	Equipment needed
LINE1	∼700,00 copies, which relates to ∼17% in human genome§	500 pg –2 µg (250 ng)	Thermal cycler, Pyrosequencer
Alu	∼1,100,00 copies, which relates to ∼11% in human genome§	500 pg –2 µg (250 ng)	Thermal cycler, Pyrosequencer
LUMA	Proportion of CpGs located within HpaII sites (5‘CCGG’3) in the human genome is 4.14% in transposable elements +3.90% in unique sequence (8.04% in total)^#^	100 −500 ng (100 ng)	Incubator, Pyrosequencer
HPLC-UV	Total 5mC in the human genome	1 –5µg (3 µg)	HPLC

§ Information taken from [37] and [38]. ^#^ Information taken from [39].

#### Estimation of LINE1 and Alu methylation using pyrosequencing of bisulfite converted DNA

Cells and colonic biopsies were assayed for global DNA methylation using the LINE1 assay described by Bollati et al. [22]. For Alu, the site of interest was the region described by Chen et al. [23], which contains an Alu repetitive element. Each sample was assayed in duplicate.

DNA was extracted from cells and tissues using standard chloroform: isoamyl alcohol extraction. Bisulfite conversion of DNA was performed using EZ DNA Methylation GoldTM kit (Zymo Research) according to the manufacturer's protocol. Briey, 250 ng of genomic DNA was incubated with CT conversion reagent at the following temperatures: 98°C for 10 min, 64°C for 2.5 hours, held at 4°C. Subsequently, DNA was transferred to a spin column, washed, desulphonated, further purified using columns, and finally eluted in a volume of 10 µl.

One microliter of bisulfite-treated DNA was added as a template in a PCR reaction containing 12.5 µL Hot Star Taq mastermix (Qiagen), 400 nM forward primer and 400 nM Biotin-labelled reverse primer in a total volume of 25 µL. The primer sequences and PCR conditions are summarized in Table 2. Amplification was carried out in a G-storm thermocycler (GRI Ltd) using the following protocol; 95°C 15 min, then 50 cycles of 95°C 15 s, annealing temperature for 30 s (55°C for LINE1 and 47°C for Alu), 72°C for 15 s, followed by 72°C for 5 min.

**Table 2:** Overview of primers and sequences for pyrosequencing assays.

Assay	Forward	Reverse	Sequencing	Sequence to analyse
LINE1	TTTTGAGTTAGGTGTGGGATATA	AAAATCAAAAAATTCCCTTTC	AGTTAGGTGTGGGATATAGT	TTC/TGTGGTGC/TGTC/TG
Alu	TTTTTTTTTAAAGGTTATG	TCTATCCCTAAAATTAAAA	TTTTTTTTTAAAGGTTATG	TC/TG

Biotin-labelled PCR products were captured with Streptavidin Sepharose beads (GE Healthcare), and made single stranded using a Pyrosequencing Vacuum Prep Tool (Qiagen). The appropriate sequencing primer (Table 2) was annealed to the single-stranded PCR product by heating to 80°C, followed by slow cooling. Pyrosequencing was carried out on a Pyromark MD system (Qiagen) and cytosine methylation was quantified using Pyro Q CpG 1.0.6 software.

#### Modified LUMA assay

The luminometric methylation assay (LUMA) was performed as described by Karimi et al. [20], with some modifications. We observed “star activity” when using the enzyme EcoRI as normalizer and therefore modified the assay using MunI as a normalization reference (Figure S1). Although, star activity of EcoRI could be prevented by using 2x Tango buffer (Figure S1B), MspI and HpaII are reported to have reduced activity in this buffer (see analysis certificates on Fermentas Life Sciences website). Moreover, methylation of CpG dinucleotides in the EcoRI restriction site impairs its cleavage activity (Figure S1E). MunI has similar cutting sites (Table 3) but exhibits better enzymatic activities when compared with EcoR1 and we observed complete DNA digestion without unspecific cuts with MunI compared with EcoRI.

**Table 3:** Overview of cutting sites for restriction enzymes.

Ezyme	Cutting sites	Methylation effect
HpaII	5′..C'CGG..3′	blocked by mCpG
MspI	5′..C'CGG..3′	no effect
MunI	5′..C'AATTG..3′	no effect
EcoRI	5′..G'AATTC..3′	impaired cleavage

After optimizing the protocol with MunI as normalizer (see validation curves and digestion pattern in Figure S1), we performed the following steps for all experiments. Briefly, 100 ng genomic DNA was digested in 2 separate 35 μL reactions with MunI/MspI or MunI/HpaII (final concentration 2.5 U/µL each; New England Biolabs) in 1x Tango Buffer (Frementas) for 16 h at 37°C. When digestion was complete, 35 µL annealing buffer (Qiagen) was added to each digestion tube. Next, the digests were distributed in triplicates (3×20 µL) in a 96-well PCR plate for analysis by pyrosequencing (PyroMark MD, Biotage). Global genome methylation (%) was calculated as follows;
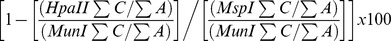
with C =  Peak height ‘C’ results, and A =  Peak height ‘A’ results.

#### Determination of 5-methylcytosine by HPLC

Frozen tissues or cell pellets were thawed and genomic DNA was isolated using standard chloroform: iso-amyl alcohol extraction. The genomic content of the nucleoside 5-methyl-2′-deoxycytidine was quantified by HPLC with UV detection. DNA digestion was performed as described by Rozhon et al. (Rozhon et al., 2008). In short, DNA was incubated overnight at 37°C with a mixture of Dnase I and nuclease P1 (both enzymes from Sigma-Aldrich, St. Louis, MO, USA). Next, nucleotide monophosphates were dephosphorylated for a further 24 h at 37°C with calf intestine alkaline phosphatase (New England Biolabs, Ipswich, MA, USA). 2′-deoxycytidine (dC) and 5-methyl-2′-deoxycytidine (5mdC) concentrations were measured with an Acquity Ultra Performance Liquid Chromatography (UPLC) system (Waters, Milford, MA, USA) which was used in high performance LC (HPLC) mode. The HPLC protocol consisted of isocratic separation using a Nucleosil SA cation exchange silica 150×4.6 mm x5 µm column (Macherey-Nagel, Düren, Germany) at a temperature of 30°C. The acidic mobile phase consisted of 50 mM ammonium acetate in 15% acetonitrile with a pH of 4.8. The flow rate was set at 0.5 mL/min and the injected sample volume was 30 µL. Samples were cooled at 4°C. UV detection was performed at 272 nm for dC and at 279 nm for 5mdC. The retention times for dC and 5mdC were 6 min 12 s and 7 min 16 s respectively. Chromatograms were analyzed using Empower 2 software (Waters). Levels of 5mdC and dC were estimated based on the corresponding standard curve which ranged from 2 to 0.008 µg/mL for 5mdC and from 20 to 0.08 µg/mL for dC (both standards from MP Biochemicals, Santa Ana, CA, USA). The relative content of 5mdC was expressed as a percentage (%5mdC) with respect to the total amount of cytosine (dC +5mdC). The technical variation of this assay is less than 3%; based on six standard replicates included in the analysis [24].

### Statistical analysis

Results are presented as mean values ± standard deviation. Differences in absolute levels of genomic methylation between the colon biopsies were analyzed by the non-parametric Wilcoxon test for matched pairs, and for analysis of the cell-based data the unpaired homoscedastic t-test was used. Correlations between assays were assessed by linear regression and by Pearson's correlations. Bland-Altman plots were prepared to investigate the agreement between pairs of assays. For these comparative analyses, absolute values were converted to percentages relative to the overall mean of each method (i.e. mean set at 100%), to generate values of similar magnitude for all of the methods. Statistical analysis was performed using SPSS v.19.0 and *P*<0.05 was considered statistically significant.

## Results

**Figure 1:**
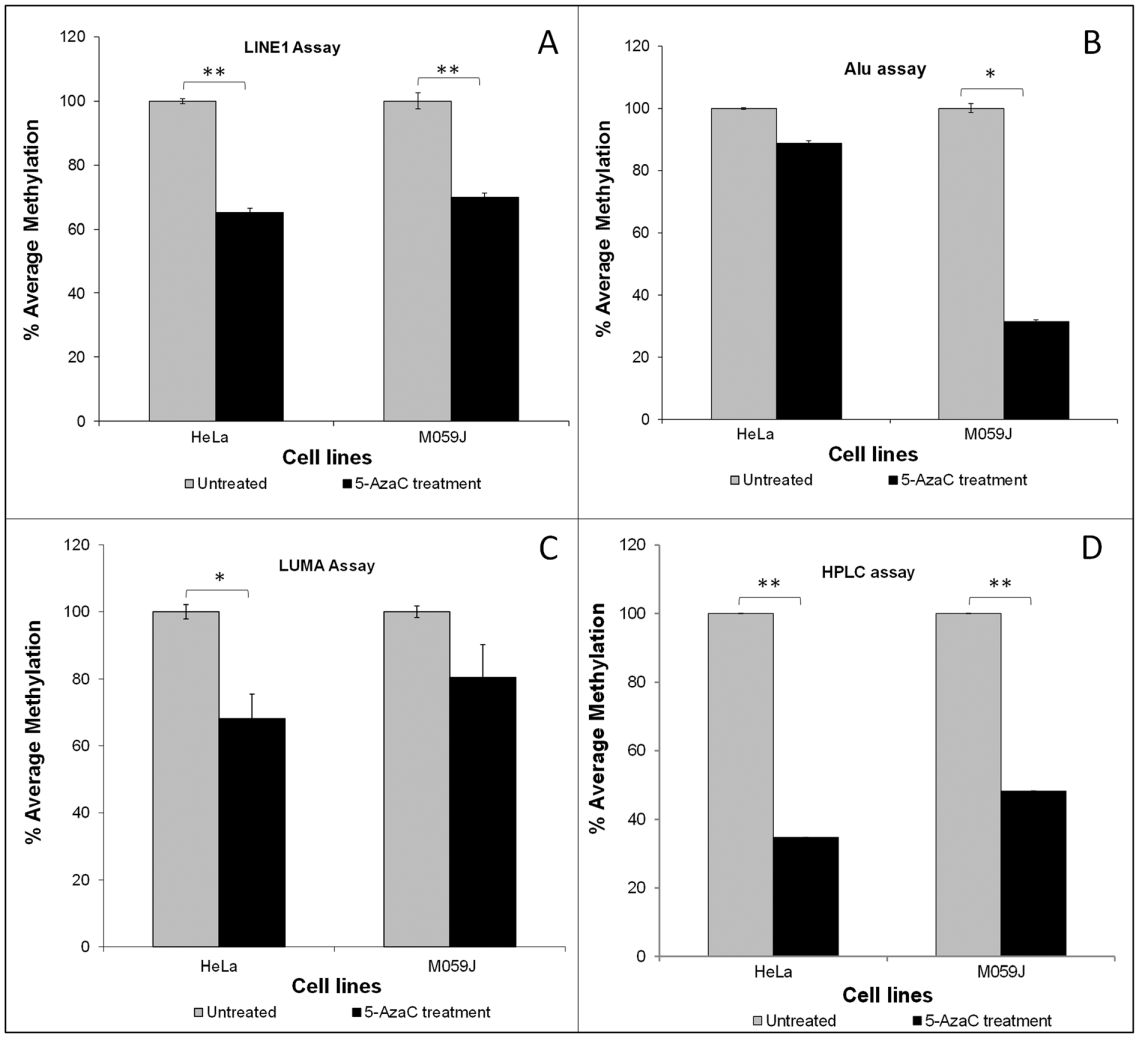
Methylation levels cell lines before and after treatment with the demethylating agent 5-AzaC. As assessed by: A) the LINE1 assay (**P≤0.001), B) the Alu assay (*P = 0.009), C) the LUMA assay (*P = 0.019), and D) the HPLC method (**P≤0.001). Data are presented as the mean % of methylation (n = 3) relative to the control (i.e. untreated cells). Error bars represent standard deviations.

**Figure 2:**
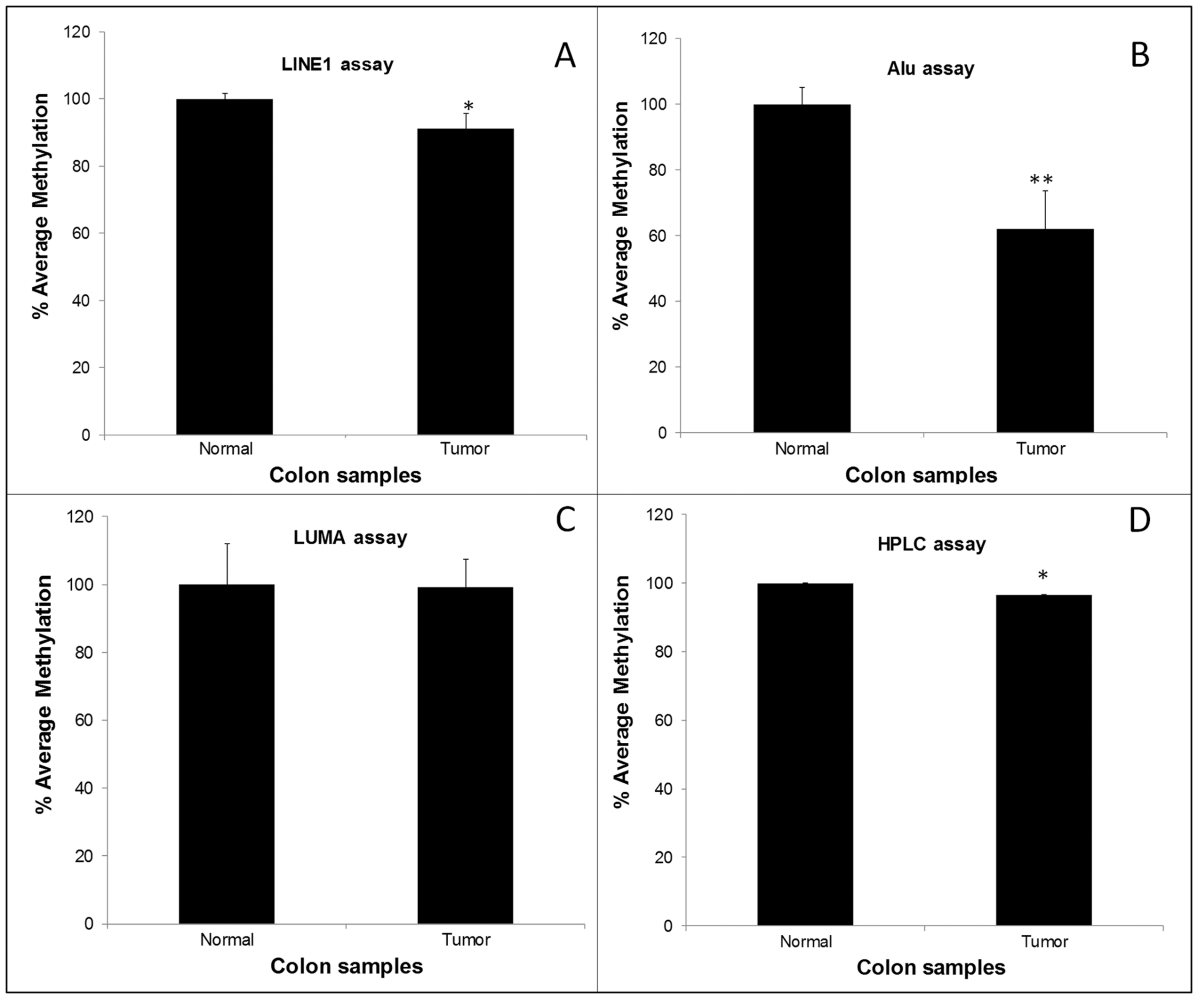
Methylation levels in tumor samples and in matched biopsies of normal colorectal mucosa. As assessed by: A) the LINE1 assay (*P = 0.013), B) the Alu assay (**P = 0.005), C) the LUMA assay, and D) the HPLC method (*P≤0.029). Data are presented as the mean % of methylation (n = 10) relative to the control (i.e. normal colon biopsies). Error bars represent standard deviations.

An overview of the absolute values for measures of genomic DNA methylation in the human cells and tissues obtained using the 4 different techniques tested in this study is given in Table 4. As expected, use of Alu, LINE1 and HPLC approaches showed reduced levels of global methylation in both cell lines after 5-azacytidine (5-AzaC) treatment (Table 4 and Figure 1). This reduction was apparent with all 4 analytical approaches, but was significant (P<0.05) only for LINE1, Alu, and HPLC approaches for M059J cells and for LINE1, LUMA and HPLC for HeLa cells (Table 1 and Figure 1). Estimates of global DNA methylation obtained with HPLC, LINE1 and Alu were lower in colorectal tumor tissue than in paired normal tissue, whereas there was no detectable difference between tissue types when assayed by the LUMA method (Table 4 and Figure 2). Because each assay measures a different variable, it is difficult to compare absolute values. Therefore, we normalized the values for controls (i.e. the untreated cells and the normal colon tissue) to 100% for each assay and recalculated the other values relative to these controls. The results for the human cells are shown in Figure 1 and for the colon biopsies in Figure 2. In both cases the HPLC and LINE-1 methods showed the smallest variation between samples, while higher variation was seen for Alu and LUMA methods (see Table 4 for CV%).

**Table 4:** Absolute values for global DNA methylation estimated by 4 different methods.

Sample	Condition/treatment	LINE1	Alu	LUMA	HPLC
		(data presented as mean % methylation ± SEM§, plus (%CV))			
Colon biopsies	Normal	71.63±0.49* (2.15%)	53.08±1.59** (9.49%)	34.76±3.83 (34.82%)	3.24±0.05* (4.53%)
	Tumor	65.38±1.39* (6.71%)	33.02±3.62** (34.66%)	34.50±2.61 (23.90%)	3.13±0.06* (5.90%)
HeLa cells	Untreated	50.21±0.92** (3.16%)	5.25±0.15 (4.83%)	52.79±1.23 (4.05%)	2.02±0.03** (2.06%)
	5-AzaC treated	32.77±0.21** (1.14%)	4.66±0.38 (14.28%)	35.98±4.22 (20.31%)	0.70±0.04** (7.07%)
M059J cells	Untreated	46.13±0.45** (1.68%)	6.09±0.82* (23.31%)	50.75±1.00 (3.40%)	1.89±0.01** (0.96%)
	5-AzaC treated	32.27±0.51** (2.76%)	1.91±0.29* (26.24%)	40.84±5.59 (23.70%)	0.91±0.02** (4.26%)

§ Data for measurements on DNA from colon biopsies are presented as mean % methylation for 10 paired samples of normal and tumor tissue (± standard error of the mean (SEM)). Data from analysis of DNA from the cells lines represent means of 3 technical replicates (± SEM).

NB: The nature of the measurements is different for each of the assays so direct comparison of the methylation percentages between assays cannot be made.

* *P*<0.03 and **P≤0.009; non-parametric Wilcoxon matched-pair test was used for comparison of normal vs. tumor tissue for colon biopsies, and unpaired homoscedastic t-test was used to test for significance between 5-AzaC- treated and untreated cells (each cell line analyzed separately).

Concerning technical replicates, the overall variation (expressed as CV%) between the replicates of the treated cells was 2.5% for LINE1, 18.5% for Alu, 13.8% for LUMA, and 2.2% for HPLC based measurements. For the colon biopsies, the overall variation (expressed as CV%) between the replicate measurements was 1.1% for LINE1, 6.7% for Alu, and 8.1% for LUMA. No technical replicates were measured via HPLC due to low amount of DNA available from the colon biopsies. However, the 5 µg/L dC and 0.5 µg/L 5mdC standards were measured five times distributed throughout the HPLC run, and the CV between these replicates was 0.5% and 2.6%, respectively. Earlier, we determined that the intra-individual sample variation of the HPLC assay is less than 10% (i.e. CV% = 3.5–7.5% between 6 measurements of the same sample, determined on different days, with 3–4 replicates in each run; unpublished data).

**Figure 3:**
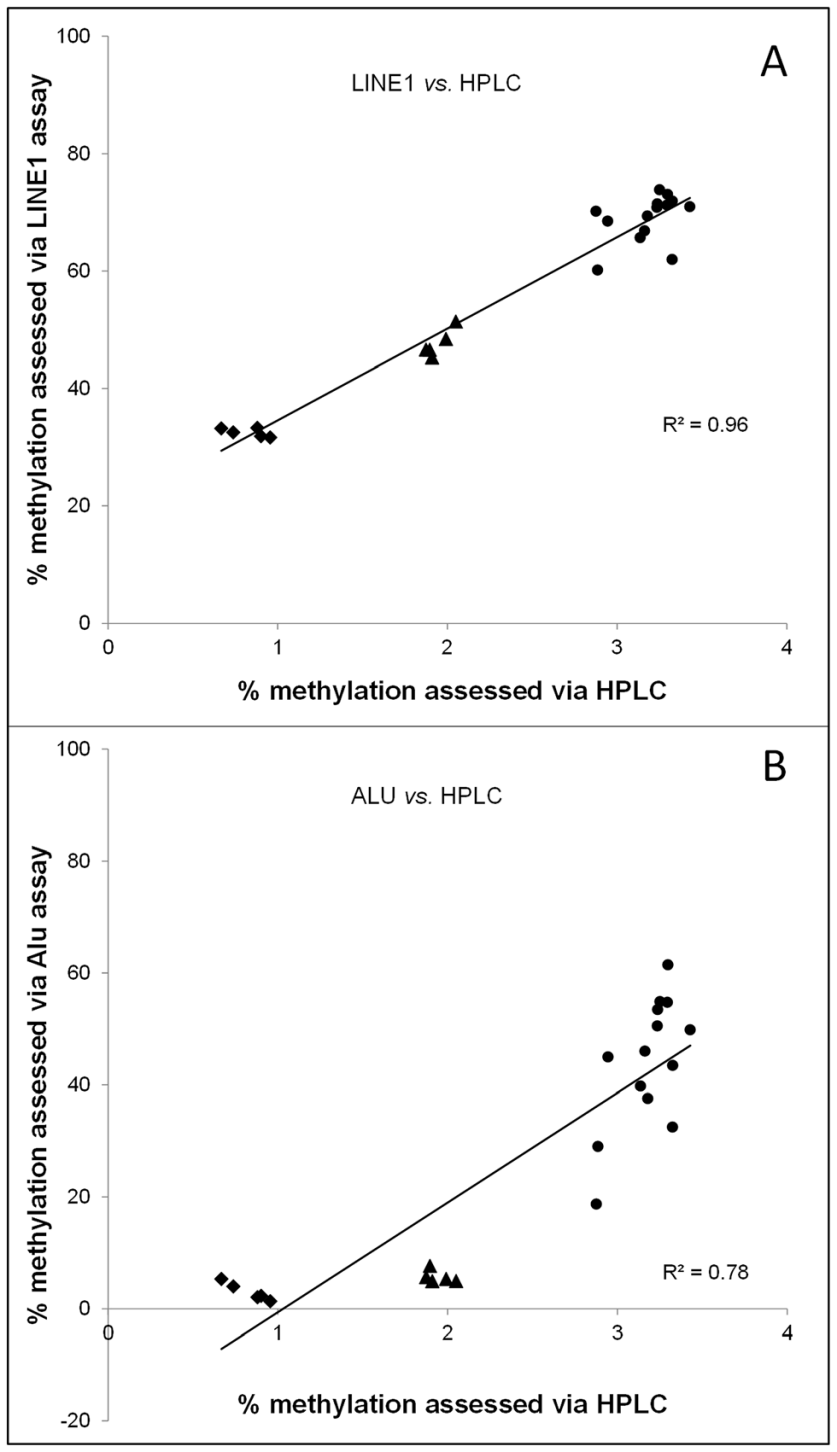
Linear regressions showing the relationships between assays. Using both data from the cell lines (⧫ 5-AzaC treated or ▴ untreated) and the colon tissues (•), significant linear associations were observed A) between the HPLC method and the LINE1 assay and B) HPLC method versus the Alu assay. Absolute values were used for these analyses.

Methylation estimates obtained using LINE1 for the cells and colon biopsies corresponded best with data measured via the gold standard HPLC approach (R^2^ = 0.96, *P*<0.001; Figure 3A). Although the correlation was weaker, the estimates obtained using the Alu assay correlated significantly with the HPLC estimates (R^2^ = 0.78, *P*<0.001; Figure 3B). In contrast, there was no correlation between estimates of global DNA methylation obtained by LUMA and those with HPLC (R^2^ = 0.04).

**Figure 4:**
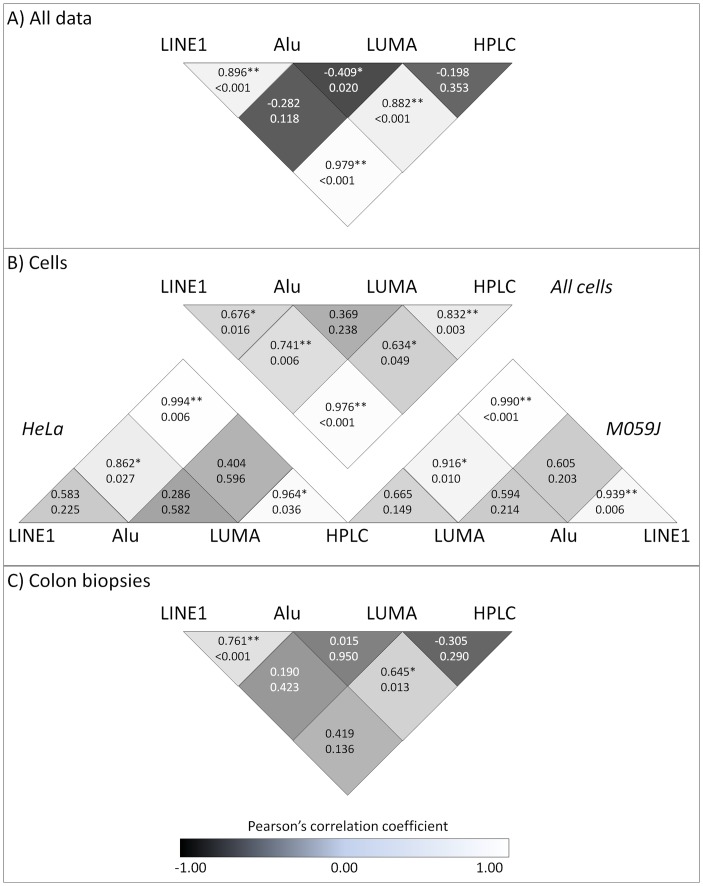
Correlation coefficients for the estimates of global DNA methylation as quantified by the various methods. A) Pearson correlations for all data combined (n = 32), B) Pearson correlation coefficients when using the data from the cells (n = 6/cell type), and C) Pearson correlations for the data from the colon biopsies (n = 20).

Figure 4 shows the outcomes of Pearson correlation analyses for all combinations of the 4 methylation assays. When considering all data together, estimates for the LINE1 and Alu assays correlated significantly with the HPLC method and with each other (Figure 4A). Next we analyzed the data for each biological source i.e. cells (HeLa and M059J) and colon biopsies separately. For the cell lines, correlations between estimates of methylation by the LINE1 and HPLC methods were positive, high and stable when data for both cell lines were pooled and when each cell line was considered separately (Figure 4B). Correlations between other pair of methods were weaker overall and, in some cases, for example Alu vs. LUMA were not significant (P>0.05) in any of the comparisons. For the colon biopsies alone, the strongest (and significant (P<0.05)) correlations were between Alu and LINE1 and Alu and HPLC. None of the other comparisons produced significant correlations (Figure 4C).

**Figure 5:**
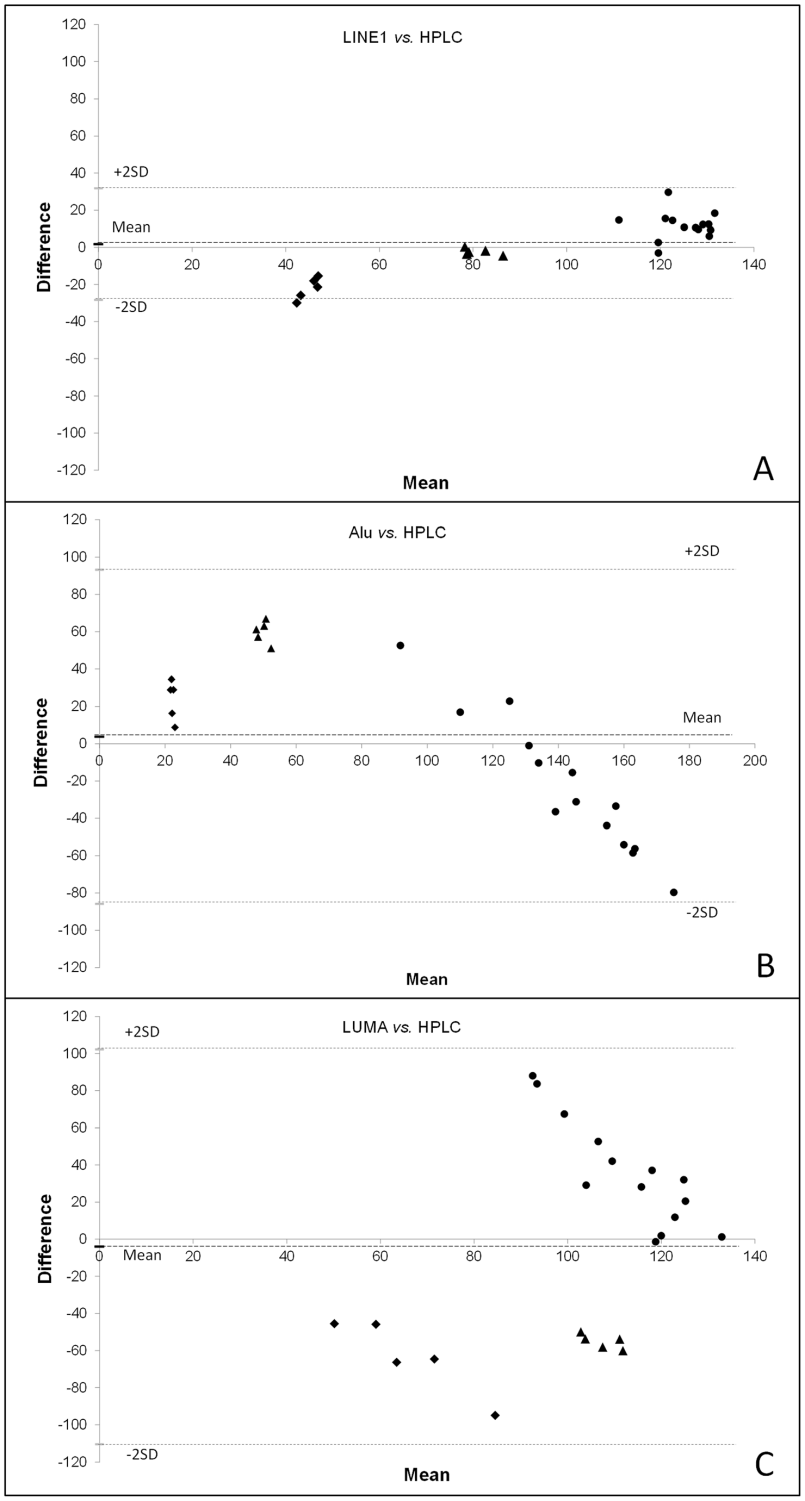
Bland-Altman plots representing the agreement of each of the surrogate assays. A) LINE1, B) Alu, and C) LUMA, with the gold standard HPLC method. The differences between the two methods (HPLC – second assay) were plotted against the averages of the two measurements, using both data from the cell lines (⧫ 5-AzaC treated or ▴ untreated) and the colon tissues (•). Horizontal lines were drawn at the mean difference, and at the limits of agreement, which are defined as the mean difference plus or minus two times the standard deviation of the differences. For these comparative analyses, absolute values were converted to percentages relative to the overall mean of each method (i.e. mean set at 100%), in order to acquire values of similar magnitude for all of the methods.

In further investigation of the agreement between results from each of the assays, Bland-Altman plots were prepared in which the differences between assays (HPLC – second assay) were plotted against the mean of the two measurements. Horizontal lines were drawn at the mean difference, and at the limits of agreement, which were defined as the mean difference plus or minus two times the standard deviation of the differences. Overall there was little bias (i.e. average difference) between estimates obtained using the LINE1 assay versus HPLC (Figure 5A) and mean differences were also low for Alu (Figure 5B) and LUMA (Figure 5C). However, observed differences for individual samples between HPLC and Alu or LUMA were much greater than for LINE1 compared to HPLC. Bland-Altman plots can also reveal potential systematic bias between assays and, for both LINE1 and Alu, there is incidence of such systematic error, which appears to be proportional to the absolute value for global DNA methylation. Although in all cases the error was relatively small for LINE1, LINE1 overestimated DNA methylation compared with HPLC when methylation levels were relatively low, and underestimated them compared to HPLC when methylation levels were higher (Figure 5A). For Alu the opposite effect was observed: relatively low levels of global DNA methylation were underestimated by the Alu assay compared with HPLC, while samples with higher methylation levels were overestimated by the Alu assay (Figure 5B). As illustrated in Figure 5C, there was poor agreement between estimates of methylation obtained by LUMA and those by HPLC. With LUMA the error (difference from HPLC estimates) increased with increasing global methylation levels, resulting in both over- or underestimation depending on the specific sample.

## Discussion

In mammals, changes in the pattern of DNA methylation across the genome are observed during development and ageing and in many pathological conditions. In addition, methylation patterns are altered by dietary and other environmental exposures throughout the life-course [25], [26]. Assessment of global DNA methylation is important since the extent of methylation can be associated with functional consequences e.g. increased mutational events and genomic instability or altered gene expression [13], [27]. Although 5mC is the major modification of cytosine in mammalian tissues, it has been shown recently that 5-hydroxymethylcytosine also occurs in mammalian DNA and may be an intermediate in the demethylation of 5mC [28]. In this study we compared estimates of global DNA methylation using 3 widely used surrogate assays (LINE1, Alu and LUMA) with each other and with estimates obtained using the gold standard HPLC. The latter is a robust, highly-reproducible methodology which provides estimates of the density of 5-methyl–2′-deoxycytidine (5mdC) in total DNA and is often expressed relative to the total amount of 2′-deoxycytidine (5mdC + dC). However, this assay requires a relatively large amount of DNA (∼1–5 µg) and the necessary equipment is not always available. Widely used surrogate assays include i) those which estimate the methylation of repetitive elements in DNA e.g. LINE1 and Alu (quantitatively major genomic domains for DNA methylation) or ii) those such as LUMA which employ parallel digestion of DNA with isoschizomers which recognize the same sequence but cut differentially according to the methylation state of a cytosine residue in the target sequence. All 3 of these techniques require relatively small amounts of starting material (∼100–500 ng DNA) and can be used as medium throughput assays suitable for large numbers of samples. In addition, all 3 of these surrogate assays cannot distinguish between 5mC and 5hmC and quantify the sum of both modifications [29], [30]. In most cases, 5hmC is a minor component of total methylated cytosine in DNA, but we have shown recently that the 5hmC:5mC ratio in brain regions of mice can range from 0.15–0.30 [31]. However, the comparability of data obtained by the various surrogate assays and by HPLC is poorly understood and failure to recognize this research gap may contribute to misinterpretation of study outcomes and to apparent contradictions between studies. Weisenberger et al. have studied comparisons between HPLC, LINE1 and Alu assays for assessment of global DNA methylation using the MethylLight method [32]. Recently, Wu *et* *al.* reported a comparison of estimates of global methylation using 3 assays for repetitive elements LINE1, Alu and Sat2 (Satellite 2– located as tandem repeats in the pericentromic and juxtacentromic heterochromatin of most chromosomes) using MethyLight assays, for LUMA and for the ^3^H-methyl acceptance assay in 4 different human blood cell types [33]. Our present study extends consideration of this issue to human tissues, uses the fast, easy but reliable, reproducible and more quantitative pyrosequencing approach instead of the MethyLight approach [34], and includes the use of the gold standard HPLC assay for global DNA methylation.

### Detection of biologically-important differences in global DNA methylation by each assay

Treatment with the demethylating agent 5-azacytidine (5-AzaC) produced a similarly large (60–65%) reduction in global methylation of DNA from both HeLa and M059J cells as measured by HPLC. All 3 surrogate assays detected lower methylation in 5-AzaC-treated cells but the degree of hypomethylation detected differed between assays and, in some cases, between cell lines (Figure 1). With the exception of the Alu assay of 5-AzaC-treated M059J cells, none of the surrogate assays revealed nearly as much genomic hypomethylation as was observed using HPLC (Figure 1).

For 3 decades, it has been recognized that human tumors are hypomethylated when compared with the corresponding normal tissue [5], [13], [35] and, in the present study, using HPLC we observed the expected lower methylation in colorectal tumor than in matched normal colon (Figure 2). A similar inter-tissue difference in global DNA methylation was detected by the LINE1 assay but the degree of hypomethylation in tumor appeared relatively greater when assessed by Alu. In contrast, the LUMA approach was unable to detect a difference in methylation between normal and tumor tissue (Figure 2). These observations show that some surrogate assays, notably LINE1, can detect biologically-important differences in global DNA methylation when these methylation changes are distributed widely across the genome.

### Correlations between assays and comparison with the gold standard HPLC

Weisenberger et al. previously showed strong correlations between global methylation levels in LINE1, Alu and Sat2 measured via MethylLight-based methods versus HPLC method [32]. For the comparisons made here, estimates of global methylation obtained using LINE1 for pooled data from both cell lines and from tumor and matched normal colon tissue correlated most strongly (R^2^ = 0.96) with those obtained by HPLC (Figure 3A). This was confirmed by the Bland-Altman analysis (Fig. 5A). Importantly, the latter showed small potential biases in LINE1-derived estimates which appeared to be slightly inflated when absolute DNA methylation levels were relatively low and somewhat underestimated at higher methylation levels (Figure 5A). This apparent bias is most likely due to the relative importance of LINE1 as a contributor to global methylation under different circumstances. Across all cells tissues, Alu-derived estimates of global methylation were quite strongly correlated (P<0.001) with those from HPLC assays (Figure 3B), but Bland-Altman analysis revealed much greater divergence between approaches in estimates of DNA methylation (Figure 5B) than were apparent for LINE1. In contrast, there was no overall correlation between LUMA-derived global DNA estimates and those from HPLC (Figure 3A), although a significant correlation was apparent when data from the cells lines only were considered (Figure 3B). Interestingly, Weisenberger et al. showed that the mean of specific Alu and Sat2 measures combined led to improved correlations with HPLC measurements, and advised that this composite measure be used for MethyLight-based estimates of genomic 5mC content [32]. When combining estimates of 5mC as detected by our LINE1 assay with our Alu-based estimates, the observed correlation of LINE1 with HPLC was not improved (R^2^ = 0.89), nor when taking the overall mean of all 3 assays versus HPLC (R^2^ = 0.86).

In studies of human blood cells, Wu et al. reported relatively strong correlations (range 0.39–0.64) between estimates based on 3 repetitive elements (LINE1, Sat2 and Alu) [33], and we have observed slightly higher correlations between LINE1 and Alu for human cell lines (R = 0.68) and for human colon tissues (R = 0.76). With the exception of HeLa cells (R = 0.86), our estimates of global DNA methylation based on the LUMA approach did not correlate significantly (P>0.05) with those for LINE1. This lack of correlation was evident for the overall data, for M059J cells and for colon tissues which supports the observation by Wu et al. of low correlations (range 0.18–0.20) between LUMA and LINE1 [33]. The LUMA assay has the advantage that it can be used with DNA from various species without extra optimization. In contrast, the LINE1 and Alu assays are genome specific and separate assays are necessary for different species. However, the LUMA assay appears to be sensitive to a number of factors including quality of the original DNA isolate (DNA fragmentation may skew results) and the choice of isoschizomers used to cut DNA in a methylation-sensitive manner. Although we performed some further optimization to improve the assay including use of MunI in place of EcoRI (Figure S1), additional optimization might be needed to improve the robustness of the assay. In addition, when interpreting their data, users of the LUMA assay should consider the distribution of the target sequence for the assay (CCGG) across the genome and the extent to which the internal cytosine in this sequence is vulnerable to loss (or gain) of a methyl group in the particular circumstances under study.

A major limitation of all measures of global DNA methylation, including those investigated in this study, is that they do not provide any information about where in the genome the methylated cytosines are located. Loss or gain of methylation from regions of heterochromatin is likely to have very different biological consequences from similar changes in euchromatin. For example, an exposure might result in increases in methylation at some genomic locations which were counter-balanced by methylation losses at other loci. In such a circumstance, use of a global DNA assay could be misleading since it might indicate “no effect” whereas, in reality, there were changes in methylation across the genome each of which might be biologically important but resulted in no net change in global DNA methylation. For these reasons, the results of assays which quantify methylation at specific, and known, genomic loci are (usually) easier to interpret.

### Conclusions

We did not find compelling evidence that any of the surrogate assays for global DNA methylation investigated here (LINE1, Alu and LUMA) could be substituted with confidence for the gold standard measurement by HPLC although LINE1 seems likely to be an acceptable surrogate in many cases. Results from a recent study of global DNA methylation in murine cells and tissues showed good correlation between LINE1 methylation and total 5-methyl cytosine measured by liquid chromatography-mass spectrometry [36]. Note, however, that LINE1 contains a minority (≈17%) of all the CpGs in the human genome and the finding that LINE1 methylation can change in some circumstances, e.g. in primary prostate cancer, without a corresponding change in overall genomic methyl cytosine content [15], is evidence that LINE1 methylation changes should be interpreted with care. Moreover, alternative approaches for the measurement of LINE1 methylation can be applied. For instance, high resolution melt analysis [36], in addition to pyrosequencing, increases the accessibility of this relatively inexpensive, and high throughput surrogate assay for overall genomic DNA methylation. In conclusion, our observations show that not all surrogate assays studied here can accurately detect relatively large, biologically-important differences in genomic DNA methylation, although, in many circumstances the LINE1 assay seems to be an acceptable surrogate for the gold standard method HPLC for measurement of global genomic methylation.

## Supporting Information

Figure S1 Validation curve for *EcoR*I incubated for 4 h in 1x Tango buffer with 0, 25, 50, 75, and 100% methylated Lambda DNA (A); Star activity was observed for *EcoR*I in 1x Tango buffer, but not in 2x Tango buffer when incubated for 4 h with methylated Lambda DNA (B); Validation curve for *Mun*I incubated with 0, 25, 50, 75, and 100% methylated Lambda DNA for 4 h or 16 h (C); in 1x Tango buffer; and E) Patterns of digested unmethylated and methylated Lambda DNA for 4 h in 1x Tango buffer with *EcoR*I or *Mun*I, showing impaired cleavage of *EcoR*I on methylated Lambda DNA (D).
(TIF)
